# Metagenomic analysis of microbe-mediated vitamin metabolism in the human gut microbiome

**DOI:** 10.1186/s12864-019-5591-7

**Published:** 2019-03-12

**Authors:** Promi Das, Parizad Babaei, Jens Nielsen

**Affiliations:** 1 Department of Biology and Biological Engineering, Chalmers University of Technology, 41296 Gothenburg, Sweden; 2 Novo Nordisk Foundation Center for Biosustainability, Technical University of Denmark, 2800 Lyngby, Denmark

**Keywords:** B-vitamins, Gut metabolism, Metagenomics, Metatranscriptomics, Vitamin prototrophs, Vitamin consumers

## Abstract

**Background:**

Human gut microbial communities have been known to produce vitamins, which are subsequently absorbed by the host in the large intestine. However, the relationship between species with vitamin pathway associated functional features or their gene abundance in different states of health and disease is lacking. Here, we analyzed shotgun fecal metagenomes of individuals from four different countries for genes that are involved in vitamin biosynthetic pathways and transport mechanisms and corresponding species’ abundance.

**Results:**

We found that the prevalence of these genes were found to be distributed across the dominant phyla of gut species. The number of positive correlations were high between species harboring genes related to vitamin biosynthetic pathways and transporter mechanisms than that with either alone. Although, the range of total gene abundances remained constant across healthy populations at the global level, species composition and their presence for metabolic pathway related genes determine the abundance and functional genetic content of vitamin metabolism. Based on metatranscriptomics data, the equation between abundance of vitamin-biosynthetic enzymes and vitamin-dependent enzymes suggests that the production and utilization potential of these enzymes seems way more complex usage allocations than just mere direct linear associations.

**Conclusions:**

Our findings provide a rationale to examine and disentangle the interrelationship between B-vitamin dosage (dietary or microbe-mediated) on gut microbial members and the host, in the gut microbiota of individuals with under- or overnutrition.

**Electronic supplementary material:**

The online version of this article (10.1186/s12864-019-5591-7) contains supplementary material, which is available to authorized users.

## Background

Human gut commensals have been known to be significant producers of vitamins, which in turn serve as an essential coenzymes for a broad class of metabolic reactions. Thus, gut commensals can synthesize vitamin K_2_ as well as the water-soluble B-vitamins, such as biotin, cobalamin, folic acid, niacin, pantothenic acid, pyridoxine, riboflavin and thiamine [1–4]. Microbial species that can synthesize vitamins de novo are known as vitamin prototrophs, and species that lack biosynthetic pathways and rely on exogenous sources are known as vitamin auxotrophs. Dietary vitamins are absorbed in the small intestine, whereas most of the microbe-mediated vitamin production takes place in the large intestine and hereby produced vitamins can eventually be absorbed by the host via specialized carrier-mediated systems, except for cobalamin [2, 5]. As the process of vitamin absorption has evolved to take place at different spatial sites, it would be reasonable to expect that there is no competition for dietary vitamins between vitamin consumers and vitamin uptake transporters in the colon and the small intestine, respectively.

Systematic assessment of the vitamin biosynthetic capabilities in gut bacteria based on genomic analysis has revealed three significant features: (a) varying prevalence and distribution of biosynthetic and transporter genes in the genomes of gut commensals; (b) lack of a complete pathways in the genome for some species; and (c) pairs of species with complementary patterns of presence and absence of genes encoding enzymes for biosynthesis of a specific vitamin [6, 7]. Furthermore, gut microbial contribution to vitamin metabolism has been recognized from several mice studies between conventional and germ-free mice experiments [8–11] and whole-genome metagenomic studies [12]. Most of the studies related to gut bacterial mediated B-vitamin metabolism have investigated the overall biosynthetic capacity of these vitamins between different groups [13, 14]. However, no study so far has reported details on the relationship between vitamin prototrophs and their consumers; abundance of vitamin biosynthetic gene(s) and uptake transporter(s) using human fecal metagenomic data; and expression level of vitamin biosynthetic genes and vitamin-dependent enzymes from human fecal metatranscriptomics data.

Therefore, to gain insights into microbe-mediated vitamin metabolism from the human fecal metagenomics and metatranscriptomics datasets, research hypotheses were formulated as follows: (i) Assuming fecal metagenomics as a representative proxy for gut microbiota, are the abundances of different vitamin metabolic genes associated to the country or health status of an individual?; (ii) Assuming vitamin metabolism as an integral part of central carbon metabolism, how is the prevalence of these metabolic genes distributed across the diverse phylum?; (iii) Based on the classification of microbial species, as vitamin producers or consumers or both, what type of species pair are correlated predominantly?; (iv) Based on the available functional annotation of these genes, is it the microbial diversity or the abundance of microbial members contributing to the variation in case-control groups? and (v) Are there parallel dependencies between the expression level of vitamin biosynthetic enzymes and vitamin-dependent enzymes?.

## Results

Our analysis involved several steps: (a) Quantitative analysis of the vitamin biosynthetic and transporter related genes between different groups; (b) Charting the information of the identified genes at their corresponding species and phyla level; (c) Identification of co-occurrence patterns between vitamin producers and consumers, and using this information to gain insights into the possible metabolic correlations based on species abundance; (d) Metabolic reconstructions of genome-scale metabolic models were deployed to visualize the community-level metabolic potential of abundant gut species in diverse cohorts, and (e) Quantifying the abundance of vitamin biosynthetic enzymes and vitamin-dependent enzymes in an IBD (Inflammatory Bowel Disease) cohort in comparison to healthy controls.

### Metagenomic abundance of vitamin biosynthetic pathways and transporters in healthy individuals from different countries

To evaluate if the abundances of microbial-mediated vitamin metabolic genes are dependent on the country of origin or not, linear and logistic regression analyses, without controlling for other confounding factors, was carried out. The result suggested an association between vitamin metabolic gene abundances with healthy samples originating from China (F-statistic, *p* < 0.01, as a country variable). However, the gene abundances from healthy samples of other countries, i.e., Denmark, Spain, and USA showed no significant association to the corresponding country variable. Extraction of DNA was performed in the Beijing Genome Institute (BGI) for the samples from China, Denmark, and Spain. Although none of these studies provided diet information for the samples, it would be reasonable to speculate that the observed differences could be a diet-dependent effect (based on Western and non-Western diets).

**Fig. 1:**
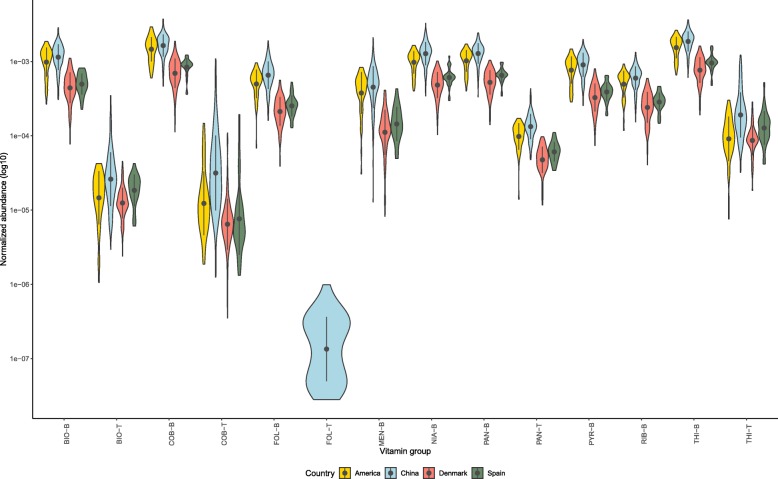
Violin plot showing total normalized abundance of genes in healthy fecal samples from four different countries (i.e. USA, China, Denmark, Spain). Microbial genes annotated to KOs were grouped into the respective type of B and K_2_ vitamins. The pointrange refers to the mean and standard deviation. The shape represents the kernel probability density of the data across different vitamin types are abbreviated as: biotin (BIO), cobalamin (COB), folate (FOL), menaquinone (MEN), niacin (NIA), pantothenate (PAN), pyridoxine (PYR), riboflavin (RIB), thiamine (THI). Suffixes that end with -B and -T are related to biosynthetic and transporter related genes respectively. [For detailed statistical results, the reader is referred to the Additional file 1: Table S11, where the asterisks indicate ns: *p* > 0.05, *: *p* < = 0.05, **: *p* < = 0.01, ***: *p* < = 0.001, ****: *p* < = 0.0001 (Mann-Whitney Wilcoxon test)]

Henceforth, we compared the normalized abundances of eight B-vitamin and menaquinone related biosynthetic and transporter genes in the healthy subjects from these different populations. On comparison of healthy samples among four different countries, we found that individuals from China had significantly higher gene abundance (Mann–Whitney test, *p* < 0.01) for each vitamin type, than that from USA, Spain, and Denmark (Fig. 1 and Additional file 1: Table S1). For each vitamin, the normalized abundance of the biosynthetic genes ranged around similar range as shown in Fig. 1 for all the healthy individuals irrespective of their origin of country. Additionally, genes involved in the biosynthetic pathways were found to be significantly higher than their corresponding transport related pathways (Mann–Whitney test, *p* < 0.01). However, it is of caution that as the bacterial vitamin transporters could belong to a diverse class of protein families [15, 16] due to their evolutionary variability, functional annotation of these transporters through bioinformatic approach remains challenging [17, 18].

### Metagenomic abundance of vitamin biosynthetic pathways and transporters in different states of health

Many functional features of gut microbiota have been shown to correlate with health and disease, and we therefore evaluated if vitamin metabolism by gut commensals is associated with the host health status. To do so, we performed similar analysis as described above between country-matched healthy controls and diseased subjects. The comparison for gene abundances included the following groups: healthy controls and the newly-onset IBD (inflammatory bowel disease) subjects from USA [14]; healthy controls and T2D (type 2 diabetes) subjects from China [13]; and healthy controls and IBD subjects under clinical remission from Spain [19]. The results suggested that gene abundances from T2D subjects in China (as a diagnosed variable) showed an association for vitamin metabolism (F-statistic, *p* < 0.01), while the gene abundances from IBD subjects from USA and Spain showed no significant association with disease status.

**Fig. 2:**
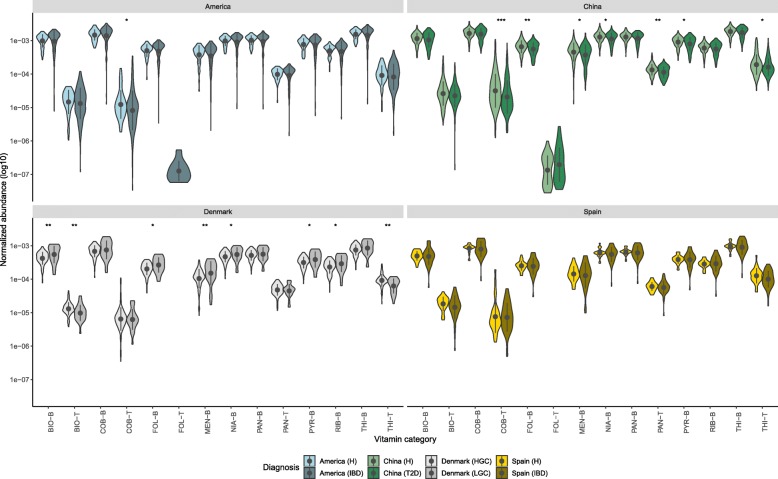
Violin plot showing total normalized abundance of genes in fecal samples from four different countries (i.e. USA, China, Denmark, Spain) under different states of health and disease. Microbial genes annotated to KOs were grouped into the respective type of B and K_2_ vitamins. The pointrange refers to the mean and standard deviation. The shape represents the kernel probability density of the data across different vitamin types are abbreviated as: biotin (BIO), cobalamin (COB), folate (FOL), menaquinone (MEN), niacin (NIA), pantothenate (PAN), pyridoxine (PYR), riboflavin (RIB), thiamine (THI). Suffixes that end with -B and -T are related to biosynthetic and transporter related genes respectively. The asterisks on the top indicate ns: *p* > 0.05, *: *p* < = 0.05, **: *p* < = 0.01, ***: *p* < = 0.001, ****: *p* < = 0.0001 (Mann-Whitney Wilcoxon test)

Comparing the gene abundances between the country-matched groups, we found that the T2D subjects from the Chinese cohort showed substantial and significant difference for all the vitamin types associated with biosynthetic enzymes and transporters (except for biotin and riboflavin) in comparison with the country-matched healthy controls (Mann–Whitney test, *p* < 0.01) (Fig. 2). On the other hand, IBD subjects (both onset and clinical remission ones) were not found to be significantly different concerning vitamin metabolism when compared to their respective country-matched healthy controls. To validate our analytical method, we tested and found that our findings were consistent with what has been reported earlier [20]. Henceforward, data for healthy subjects were used to get insight into how these genes map to bacteria from different phyla.

### Phylogenetic origin of vitamin pathway genes

**Fig. 3:**
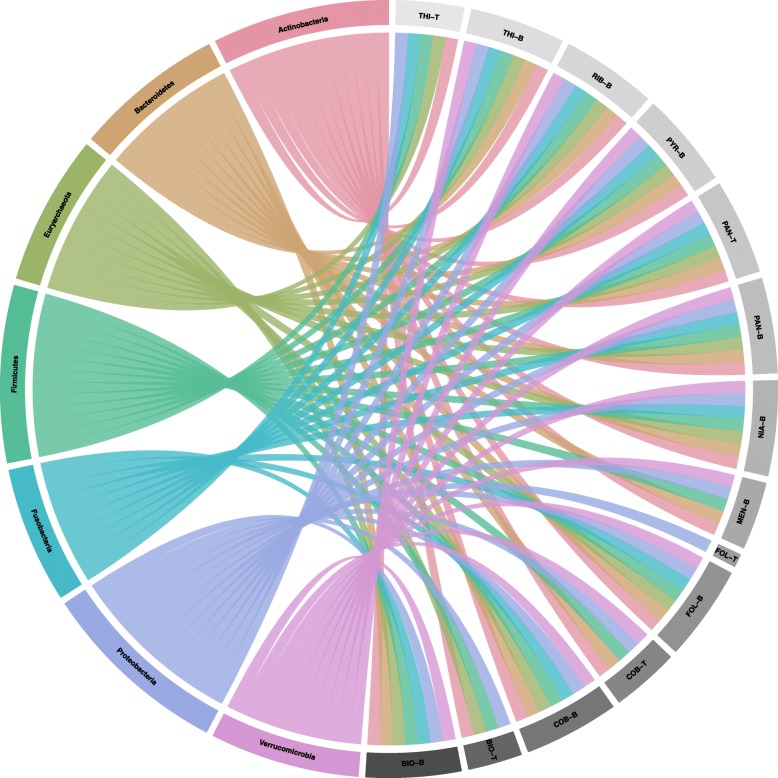
Circular plot with links representing the prevalence of vitamin metabolism among different phyla of human gut microbiota. Each phylum is colored and each type of vitamin phenotype is shown in grey. The vitamin types are abbreviated as: biotin (BIO), cobalamin (COB), folate (FOL), menaquinone (MEN), niacin (NIA), pantothenate (PAN), pyridoxine (PYR), riboflavin (RIB), thiamine (THI). Suffixes that end with -B and -T are related to biosynthetic and transporter related genes respectively

To compare the taxonomic profile of vitamin metabolic genes (biosynthesis and transporter genes) at phylum or species level, each of the mapped gene identifiers were used to retrieve the lineage information from UniProt database. In Fig. 3, we show how vitamin metabolic genes are prevalent across different phyla, which might suggest vitamin metabolism being as a part of core function in gut microbial metabolic modules. Additional file 2: Figure S1 shows a network of associations between species with mapped vitamin-related gene and its corresponding phylum. This figure reveals that the vitamin related pathways are mostly found in the phylum Firmicutes with about 49%, followed by Proteobacteria with about 19%, Bacteroidetes with about 14% and Actinobacteria with about 13%. Additional file 3: Figure S2 shows a network of associations between vitamin biosynthetic capabilities and phylum with species information. For every vitamin type, the number of species having prevalence of a biosynthetic pathway seemed to be higher than that of species with transporter mechanisms. Among the various vitamin transporters, species encoding thiamine transporter dominated in contrast to folate transporter being the lowest in number. However, from Additional file 3: Figure S2, it also becomes apparent that there are three types of species that differ with respect to functional characteristics related to vitamin metabolism, i.e. vitamin genes related to biosynthesis or transporters or both. Based on this, we categorized 171 microbial species as Producer (P), Consumer (C) and Producer-Consumer (Dual) for three specific vitamins (biotin, cobalamin, and thiamine) in the following analysis.

### Network association between vitamin prototrophs and vitamin consumers

Given a hypothetical situation where there is a direct dependence between public-good-producing bacteria and its consumer, it is expected to observe a stronger association of their coexistence at the level of species abundance. Based on the presence or absence of genes encoding proteins required in vitamin biosynthetic pathways or transporter mechanisms or both, each microbial species was assigned as either vitamin prototrophs (producers) or vitamin consumers (consumers) or producer-consumer (dual) respectively for every vitamin type. Therefore, to estimate interactions between vitamin prototrophs and consumers, we performed a correlational analysis between species with all possible phenotypes. Of total 7021 possible pairs of species combinations (171 species (675 strains) considered), 2030 (i.e. 28.9%) and 590 (i.e. 8%) were significantly positive and negative correlated, respectively (Mann–Whitney test, *p* < 0.01). Among the positive correlations, with a correlation coefficient greater than 0.7, we observed that a pair of species originating from the same phylum could coexist, probably due to their evolutionary similarities. However, we also observed a similar level of coexistence for a pair of species originating from different phyla. For example, species from two different phyla, *Lactobacillus acidophilus* (Firmicutes) and *Citrobacter koseri* (Proteobacteria) had a high and positive correlation coefficient r = 0.81.

Based on the high prevalence of species with vitamin metabolic pathways among different phyla as derived from Fig. 3, we focused our next correlational analysis (using similar approached as described above) to the most prevalent three B-vitamins among microbial species, i.e. biotin, cobalamin, and thiamine involving both biosynthetic and transporter related genes respectively to estimate interactions between these specific vitamin prototrophs and consumers. Contrary to our hypothesis, we found that species with dual functional traits (i.e. containing genes for both biosynthesis and transport of the same vitamin) contributed to a notable fraction of positive correlations among the possible phenotypes (Additional file 4: Figure S3). On the other hand, the co-occurrence of species with single phenotype (i.e., either producer/P or consumer/C) and dual phenotypes belonged to both positive and negative correlations. These observations hint that the species with dual phenotypes could drive stronger relationships than the ones with either limited or less functional traits. It is well-known, that communities of species with multiple functional features respond well to changes in the environment, thereby influencing the net outcome of a community assembly [21–23]

### Genome annotation coverage of biosynthetic pathways

Next, we sought to visualize the presence and absence of vitamin pathway related reactions in each cohort to gain insights into how conserved each pathway is, across different populations. As depicted in Additional file 5: Figure S4, riboflavin seems to be well represented and conserved among all species across all the four cohorts regardless of the health status. This conservation could also be due to the limited number of reactions involved in riboflavin pathway compared to other pathways. Contrarily, menaquinone or vitamin K_2_, has the least number of species with its biosynthesis pathway present. Perhaps, human microbiome might have evolved to have a reduced dependence of menaquinone production from the gut microbiota as proposed earlier [24]. In case of folate, the majority of the reactions involved in its biosynthesis were observed to be present except for four reactions which concerned interconversions of tetrahydrofolate, dihydrofolate, and folate using NADH or NADPH as cofactors and one ATP-forming reaction carried out by folylpolyglutamate synthase enzyme. Biotin and thiamine appeared to be vitamins with a major part of their biosynthetic reactions with their annotations missing. These are related to fatty acid metabolism and the upstream part of the biotin synthesis pathway provides a long-chain acyl as the starting point for the biosynthesis. In case of thiamine, three phosphatase reactions involving interconversion of thiamine monophosphate, −diphosphate, −triphosphate and thiamine were absent. For the rest of the vitamins, the incompleteness of their biosynthesis pathway was observed for many species, which is in line with previous studies as it has been suggested that vitamin biosynthesis could be carried out as complementary tasks between several bacteria, each harboring a part of the pathway [25].

**Fig. 4:**
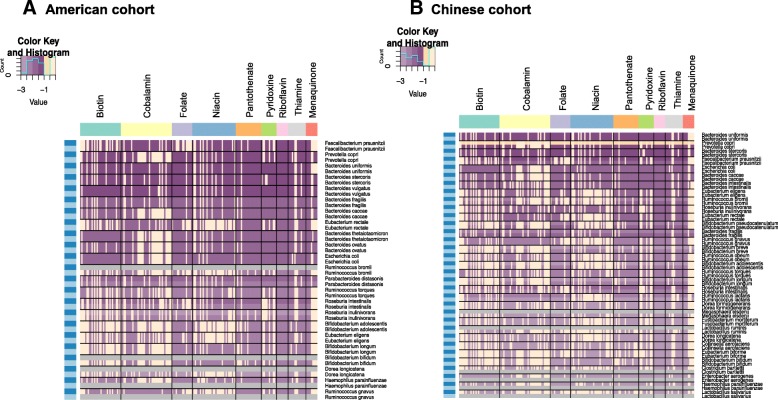
Heatmap showing relative gene abundance of reactions associated with gene annotations of vitamin biosynthetic pathways in genome-scale metabolic models (GEMs) based on species abundance of abundant gut bacteria (a) American cohort and (b) Chinese cohort. The x-axis represents the reaction IDs associated to each vitamin pathway and on the y-axis the list of abundant gut bacteria are shown. Light blue and dark blue (on the left) represents healthy and diseased of associated reaction abundance to each microbial species (on the right) respectively. Grey color in every row represents absence of species in the cohort and the purple color key (dark to light color) represents abundance from high to low values

Furthermore, for the American and Chinese cohort, we investigated the presence and absence of reactions in the biosynthesis pathway of each vitamin considering the abundance of bacterial species. As depicted in Fig. 4a, it is noticeable that species with biosynthetic reactions of riboflavin, pantothenate, and pyridoxine have relatively higher coverage when compared to the other vitamin types. It is also to be noted that species from genera Bacteroides, namely *B. uniformis*, *B. stercoris*, *B. vulgatus*, *B. fragilis*, and *B. caccae* are among the most abundant species with relatively high coverage of biosynthetic capabilities for all vitamins. Although the overall coverage of vitamin biosynthetic pathways is rather high, the same trend is not observable in case of biotin, cobalamin, and niacin which have incomplete pathways in many cases.

For the Chinese cohort (Fig. 4b), we noticed that the abundant species profile differs from that of the American cohort, possibly reflecting a population-specific (which is mostly due to country variation) gut microbiota. It is again evident from this that Bacteroides species have most of the vitamin biosynthesis pathways covered except for cobalamin due to differences at the species level. Besides, in the Chinese population, there are many species from Firmicutes amongst the abundant ones, which suggest that based on the Bacteroidetes/Firmicutes ratio there may be a change in abundance of vitamin related genes.

### Expression level of vitamin biosynthetic enzymes and vitamin-dependent enzymes in an IBD cohort

**Fig. 5:**
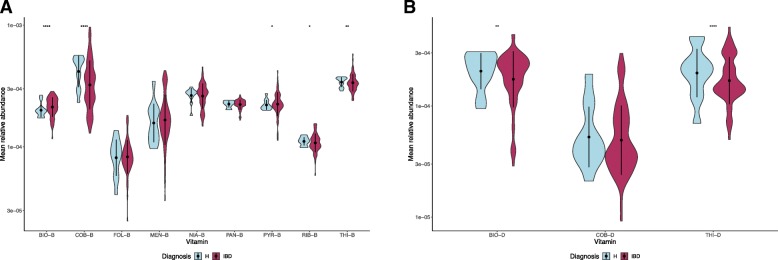
Violin plot showing mean relative abundance of (a) vitamin-biosynthetic genes and (b) vitamin-dependent genes in fecal samples from American cohort. Microbial genes annotated to EC numbers were grouped into the respective type of B and K_2_ vitamins. The pointrange refers to the mean and standard deviation. The shape represents the kernel probability density of the data across different vitamin types are abbreviated as: biotin (BIO), cobalamin (COB), folate (FOL), menaquinone (MEN), niacin (NIA), pantothenate (PAN), pyridoxine (PYR), riboflavin (RIB), thiamine (THI). Suffixes that end with -B are biosynthetic related enzymes. The asterisks on the top indicate ns: *p* > 0.05, *: *p* < = 0.05, **: *p* < = 0.01, ***: *p* < = 0.001, ****: *p* < = 0.0001 (Mann-Whitney Wilcoxon test)

Based on the availability of metatranscriptomics dataset from the American cohort analyzed above, we sought to investigate the mRNA sequences of microbe-mediated vitamin related enzyme as computed by HUMAnN2 [26]. Functional profiling of mRNA normalized features (i.e., vitamin biosynthesis) in this cohort reveals that there is a low abundance of cobalamin and thiamine biosynthesis related enzymatic genes expressed in the IBD samples compared to healthy controls (Mann–Whitney test, *p* < 0.01) (Fig. 5a). These findings were consistent with a similar pattern observed in a previous study where B-vitamins were measured to be lower in plasma samples of IBD subjects [27–29].

However, the expression level of biotin biosynthetic enzymes was found to be significantly higher in the IBD subjects compared to healthy controls (Mann–Whitney test, *p* < 0.01). To examine this, we inspected whether the abundance of biotin synthesizers were significantly abundant or not in the IBD subjects than that of the healthy controls (especially, for the coordinated set of samples where the metagenomics and metatranscriptomics were carried out). Interestingly, we found that species with only biosynthetic pathways for biotin were significantly more abundant in the IBD subjects whereas species with biotin transporters were significantly less abundant in the IBD subjects compared to healthy controls (Additional file 6: Figure S5).

These observations once again highlight the significance of the microbial contribution to human health status. Furthermore, we analyzed the expression level of these vitamin-dependent enzymes to discern the connection between their synthesis and usage. We found that the abundance of biotin-dependent enzymes had an inreverse pattern to that of their biosynthesis level, whereas, thiamine utilization potential as well as its biosynthetic potential were less abundant in IBD subjects when compared to the healthy controls (Fig. 5b) (Mann–Whitney test, *p* < 0.01). Variation in vitamin synthesis and utilization level among these three vitamins is likely to reflect the complexity of the gut microbiota towards resource allocation and reuse of enzymes.

## Discussion

Here we used whole-genome metagenomic sequences and performed bioinformatic analyses to gain an understanding of the microbe-mediated vitamin biosynthetic pathways and transport mechanism of the human gut microbiota. Fecal metagenomes sampled from four different countries were analyzed together to improve the robustness of our predictions at a statistical level. This provided insights into how a population-specific microbiome or a microbiome-specific population differs with respect to vitamin metabolism. Our approach of computational method enabled us to analyze a targeted pathway with an identification of the related KEGG Orthologies. We focused on vitamin metabolism of human gut microbiota as it might be conserved across distantly related microbial species, potentially because they are an integral part of the carbon metabolic network.

Based on the hypotheses and the analyses performed on fecal metagenomics from four different countries, we highlight the following findings to assess ecological relationship between vitamin prototrophs and consumers: (a) B and K_2_ vitamin metabolism is prevalent across most abundant phyla, i.e., Bacteroidetes, Firmicutes, Actinobacteria, Proteobacteria, Fusobacteria, Verrucomicrobia, and Euryarchaeota; (b) Healthy individuals from China had a significantly higher abundance of these metabolic genes in comparison to healthy individuals from Western countries such as Denmark, Spain, and USA; (c) At metagenomic level, T2D subjects had significant changes in the gene abundance profile related to microbe-mediated vitamin metabolism; whereas only a part of B-vitamins has been found to differ in their abundance between healthy controls and IBD subjects; (d) Dual microbial species (i.e. containing genes for both biosynthesis and transport of the same vitamin) tend to co-occur in higher proportion than that with either traits; (e) Estimation of vitamin-specific pathways from metabolic reconstructions for abundant microbes reveal contributions to ecosystem-scale metabolic processes; (f) Evaluation of their gene expression levels in an IBD cohort reveals the significance of microbial contribution towards the host. Overall, our findings provide valuable new insights related to vitamin field of metabolism in the human gut microbiota.

Analogous to the food webs, the equation between vitamin producers and its consumers cannot be directly comparable as that of predator-prey relationships for “public good” (vitamin), as there could be a combination of factors driving the coexistence of two species together. Based on the species co-occurrence abundances, species with dual functional traits were found to be dominant and with a tendency to cooccur with each other. This observation is consistent with the notion that species equipped with diverse set of functional traits seems to establish resilient communities [21–23]. While the direction from correlation to causation might not hold true in every situation, we believe that these predicted positive and negative associations could imply reasons other than the parameterized variable. For instance, even though *Bacteroides thetaiotamicron* could produce biotin and *Bifidobacterium adolescentis* could uptake biotin, we found that *B. thetaiotamicron* and *B. adolescentis* were negatively correlated. As an another example, *Faecalibacterium prausnitzii* and *Roseburia inulinivorans* can produce cobalamin, but they were positively correlated [7, 30]. Experimental in vitro co-culturing of these pairs (i.e., for their co-growth evaluations) has been validated by co-occurrence network predictions [30]. Though further study into the micro-ecology of this pattern in a defined gut microbiota model is required, as a working hypothesis a major driver for co-occurrence seems to be the ability to cohabitate in a given environment. This is in agreement with the results of [31] who found that bacteria evolve and respond according to environmental changes.

Though the predictions at metagenomics level for the American cohort revealed no significant difference between healthy and IBD subjects regarding vitamin-related gene abundances, it is worth considering to probe into the expression of biosynthetic enzymes from metatranscriptomics. To profile the gut microbiome’s functional capacity about vitamins, we therefore used metagenomic functional annotation of these genes encoded in the gut microbiome, and combined this with available metatranscriptomics data for an IBD cohort. The gene expression level for vitamin related enzymes was congruent with previous clinical reports where vitamin measurements from plasma samples of IBD subjects reveal a decreased level of cobalamin and thiamine. Although we observed an increase in the expression levels for biotin in IBD subjects, we also found a pattern where species with biotin producing potential were abundant in IBD subjects. These findings suggest that it is not only the difference in species abundance that sets two groups apart, it is also the functional features of the abundant species that contributes to determining a healthy gut microbiome.

## Conclusions

Our study reveals the country-specific differences between healthy and disease (i.e., T2D and IBD) subjects for at least some of vitamin biosynthetic pathways, which highlights the role of diet and lifestyle in the correlation between human gut microbiota and the health status of the host. As the diet-derived vitamins are absorbed in the small intestine, it seems that the country-specific, microbial genetic content (vitamin metabolic genes derived from vitamin producing microorganisms) in the large intestine accounts for the observed differences in different cases as revealed through the abundant community-level metabolic reconstructions. Even if vitamin dysbiosis cannot be associated with an altered gut microbiota-mediated metabolic disease, we believe that it may well be an integral part of the bacterial central carbon metabolism, contributing among themselves and to the human host. Estimation of the contributory levels of human gut microbiota towards vitamin host absorption, could disentangle the interrelationship between the impact of B-vitamin dosage on gut microbial members and the host, especially in the gut microbiota of individuals diagnosed with under- or overnutrition and T2D. We are therefore confident that our study would enable screening for microorganisms with high potential for vitamin production. In related to screening results for synthetic communities of vitamin production, it may become possible to improve the current vitamin fortification programs in the food-microbiome industries through the administration of live or attenuated vitamin-producing bacteria into fermented foods. Such bacteria may offer a natural alternative than consuming vitamin fortified foods where their composition are above the Estimated Average Requirement (EAR) [32].

## Methods

### Cohort description

Metagenomic sequences subjected to Illumina paired end sequencing were obtained from European Nucleotide Archive at EMBL-EBI (https://www.ebi.ac.uk/ena) under the accession number PRJEB2054, PRJNA422434, PRJNA275349, and PRJNA389280.

From PRJEB2054, 14 and 25 samples were of healthy controls and IBD subjects from Spain respectively [19]. Healthy controls were recruited among family relatives of IBD patients; antibiotic treatment for at least 4 weeks before fecal sample collection was excluded. IBD subjects were in clinical remission for at least 3 months, and had stable maintenance therapy with mesalazine or azathioprine [33]. 15 and 70 samples originating from Denmark were of low-gene count and high-gene count respectively [34].

From PRJNA422434, 187 healthy controls and 172 subjects who were diagnosed with Type 2 Diabetes were analyzed. These fecal samples were sampled from Shenzhen Second People’s Hospital, Peking University Shenzhen Hospital and Medical Research Center of Guangdong General Hospital, individuals living in the south of China. Detailed clinical information for the samples is available in the supplemental information of the original article [13].

From PRJNA389280, 55 healthy controls and 223 IBD subjects were analyzed. None of the IBD subjects had history of terminal ileal resection. The samples were collected and recruited from Massachusetts General Hospital, Cedars-Sinai Medical Center, and Emory University and Cincinnati Children’s Hospital [35]. Detailed information on the criteria considered for the sample inclusion or exclusion on IBD subjects is available in the original article [14].

### Functional mapping and analysis

Vitamin related pathway abundances were calculated using an open-sourced, stand-alone functional analysis pipeline FMAP [36]. Metagenomic read sequences were mapped to the reference database using DIAMOND [37] as mapping mode. Unique hits that passed through the default parameters (i.e., e-value >1e-10 and sequence identity > 80%) of the pipeline FMAP were considered for further analysis. From the BLAST output files, the subject sequence identifiers were used for identification of taxonomic lineage and KO analysis. Gene sequences as determined by UniRef were mapped to Kyoto Encyclopedia of Genes and Genomes (KEGG) Orthologies (KOs) database [38, 39] and were binned into functional categories as listed in Additional file 7: Table S1-S9. Taxonomic lineage of the mapped hits were retrieved from the UniProt website (dated on October 2018). For every vitamin type, total normalized abundance, was computed as number of reads that have been aligned to a gene divided by total number of read counts in a sample followed by summation of all the genes constituting the pathway. Or in other words, the summation of the normalized abundances of each vitamin’s KO in each sample was defined as the total normalized abundance for each vitamin pathway.

Relative abundance of the species was calculated using MEDUSA [40] for the MetaHIT and T2D cohort. Data table with relative abundance of species and metatranscriptomics data for the HMP2 cohort were obtained from their publicly available project website [14]. Using the species abundance values, spearman correlation was applied to estimate correlations in determining the relationship between different microbial species. Pairs of species with correlation coefficient of greater than or equal to 0.4 were considered as positive correlations. Pairs of species with correlation coefficient of less than zero were considered as negative correlation. Based on the taxonomic information extracted for each of the mapped protein identifiers from the UniProt, microbial species encoding proteins required for biosynthesis of a certain vitamin but lacking transporters for it were assigned as vitamin prototrophs (producers or ‘P’). The species having the transporters but lacking the biosynthetic genes for a certain vitamin were assumed as vitamin auxotrophs (consumers or ‘C’). As well, the species containing genes for both the biosynthesis and the transport of the vitamin were assigned as dual (producer-consumer or ‘PC’).

For identification of vitamin-dependent enzymes, their enzyme commission numbers were obtained from ExPASy (listed in Additional file 7: Table S10).

### Metabolic reconstructions

In order to investigate the coverage of vitamin biosynthesis pathways, previously published repository of human gut bacterial genome-scale metabolic models were deployed [41]. These models have a GPR (gene-protein-reaction) association component which were used to translate gene profiles to reactions through logical associations. From this repository, vitamin biosynthesis profile of the overlapping species was looked in each cohort. To do so, all the KO groups related to vitamin biosynthetic reactions as described in the previous section were extracted. KEGG reactions were mapped to Kbase [42] nomenclature systems to be consistent with the reaction identifiers in the metabolic models. Following identification, a list of biochemical interconversions for each vitamin category, namely, biotin, cobalamin, folate, niacin, pantothenate, pyridoxine, riboflavin, thiamine and menaquinone were complied. Subsequently, the presence and absence of these reactions in each cohort were analyzed to see how conserved each pathway is across different populations. Furthermore, for the American and Chinese cohort, we investigated the presence and absence of the reactions in biosynthesis pathway of each vitamin considering the abundance of bacterial species. In each community, a union list of the present species was constructed and sorted according to the relative abundance of the species in the healthy cohort. Species unique to the diseased population were then added to the end of the list.

### Statistical analyses

All the statistical analysis and graphics were performed using R version 3.5.1 [43]. Country matched samples were compared into two groups for differential KO abundance testing between healthy controls and diseased subjects to avoid cross country-effects. Statistical analyses that involved two groups and more than two groups was performed by Mann-Whitney-Wilcoxon tests and Kruskal Wallis tests respectively. Bonferroni’s correction was applied to the significance level. For multivariable linear regression, categorical metadata and vitamin type abundances, samples were pooled into classifications (male/female, healthy/diseased, specific nationality/rest, etc) and significance was identified using Fisher’s exact test with multiple testing correction of *P* values.

## Additional files

Additional file 1: **Table S1.** Statistical information on comparison of vitamin gene abundances between different countries. (XLSX 13 kb)

Additional file 2: **Figure S1.** Circular plot with links representing the prevalence of vitamin metabolism among different microbial species from different phyla of human gut microbiota. (DOCX 374 kb)

Additional file 3: **Figure S2.** Circular plot with links representing the prevalence of vitamin metabolism among different microbial species from different phyla of human gut microbiota. (DOCX 3215 kb)

Additional file 4: **Figure S3.** Alluvial plot showing the relationship between the kind of correlation for each vitamin type (biotin, cobalamin and thiamine) and the possible phenotype combinations. (DOCX 3751 kb)

Additional file 5: **Figure S4.** Heatmap showing the presence and absence of reactions associated with gene annotations of vitamin biosynthetic pathways in genome-scale metabolic models (GEMs) of abundant gut bacteria. (DOCX 1813 kb)

Additional file 6: **Figure S5.** Boxplot showing total relative abundance of species with biotin biosynthesis (BIO-B) and/or biotin transporters (BIO-T) in samples from American cohort. (DOCX 21 kb)

Additional file 7: **Table S1–Table S10.** List of all the KEGG Orthologies (KOs) and Enzyme Commission (EC) numbers that were studied for each vitamin pathways. (XLSX 31 kb)

## Abbreviations

BGI: Beijing Genome Institute
BIO: Biotin
C: Consumer
COB: Cobalamin
EAR: Estimated Average Requirement
FOL: Folate
GEMs: Genome-scale metabolic models
GPR: Gene-protein-reaction
IBD: Inflammatory Bowel Disease
KEGG: Kyoto Encyclopedia of Genes and Genomes
KOs: KEGG Orthologies
MEN: Menaquinone
NIA: Niacin
P: Producer
PAN: Pantothenate
PYR: Pyridoxine
RIB: Riboflavin
T2D: Type 2 Diabetes
THI: Thiamine
