# Supplementary material for: Metagenomic analysis of microbe-mediated vitamin metabolism in the human gut microbiome
Source: BMC Genomics. 2019 Mar 12;20:208. doi: 10.1186/s12864-019-5591-7 (PMC6417177; doi:10.1186/s12864-019-5591-7)
Supplement: Supplementary file 2 — Figure S1. Circular plot with links representing the prevalence of vitamin metabolism among different microbial species from different phyla of human gut microbiota. (DOCX 374 kb) [file 12864_2019_5591_MOESM2_ESM.docx]

Figure S1. Circular plot with links representing the prevalence of vitamin metabolism among different microbial species from different phyla of human gut microbiota. Each phylum and vitamin type is colored and each microbial species is shown in grey.
