# Supplementary material for: Metagenomic analysis of microbe-mediated vitamin metabolism in the human gut microbiome
Source: BMC Genomics. 2019 Mar 12;20:208. doi: 10.1186/s12864-019-5591-7 (PMC6417177; doi:10.1186/s12864-019-5591-7)
Supplement: Supplementary file 4 — Figure S3. Alluvial plot showing the relationship between the kind of correlation for each vitamin type (biotin, cobalamin and thiamine) and the possible phenotype combinations. (DOCX 3751 kb) [file 12864_2019_5591_MOESM4_ESM.docx]

**Figure S3.** Alluvial plot showing the relationship between the kind of correlation for each vitamin type (biotin, cobalamin and thiamine) and the possible phenotype combinations. The abbreviations for P, C and Dual are ”producer”, ”consumer” and species with biosynthetic and transporter related pathways.
