# Supplementary material for: Metagenomic analysis of microbe-mediated vitamin metabolism in the human gut microbiome
Source: BMC Genomics. 2019 Mar 12;20:208. doi: 10.1186/s12864-019-5591-7 (PMC6417177; doi:10.1186/s12864-019-5591-7)
Supplement: Supplementary file 5 — Figure S4. Heatmap showing the presence and absence of reactions associated with gene annotations of vitamin biosynthetic pathways in genome-scale metabolic models (GEMs) of abundant gut bacteria. (DOCX 1813 kb) [file 12864_2019_5591_MOESM5_ESM.docx]

Biotin

Cobalamin

Folate

Niacin

Pantothenate

Pyridoxine

Riboflavin

Thiamine

Menaquinone

America

China

Denmark

Spain

**Figure S4**. Heatmap showing the presence and absence of reactions associated with gene annotations of vitamin biosynthetic pathways in genome-scale metabolic models (GEMs) of abundant gut bacteria. X-axis represents the reaction IDs associated to each vitamin pathway. Y-axis represents the list of abundant gut bacteria that are unique for health, core microbial species between groups from each country and unique for disease (as shown in a three gradient color scale under each country vertical axis). Light blue and dark blue represent absence and presence of associated reaction to each microbial species respectively.
