# Supplementary material for: Metagenomic analysis of microbe-mediated vitamin metabolism in the human gut microbiome
Source: BMC Genomics. 2019 Mar 12;20:208. doi: 10.1186/s12864-019-5591-7 (PMC6417177; doi:10.1186/s12864-019-5591-7)
Supplement: Supplementary file 6 — Figure S5. Boxplot showing total relative abundance of species with biotin biosynthesis (BIO-B) and/or biotin transporters (BIO-T) in samples from American cohort. (DOCX 21 kb) [file 12864_2019_5591_MOESM6_ESM.docx]

**Figure S5**. Boxplot showing total relative abundance of species with biotin biosynthesis (BIO-B) and/or biotin transporters (BIO-T) in samples from American cohort. The upper, middle and lower lines of the boxplot represent first quartiles, medians and third quartiles with the whiskers indicating 1.5 times inter-quartile range. The asterisks on the top indicate ns: p > 0.05, *: p <= 0.05, **: p <= 0.01, ***: p <= 0.001, ****: p <= 0.0001 (Mann-Whitney Wilcoxon test).
